# An extracellular thermo-alkali-stable laccase from *Bacillus tequilensis* SN4, with a potential to biobleach softwood pulp

**DOI:** 10.1007/s13205-014-0207-z

**Published:** 2014-03-23

**Authors:** Sonica Sondhi, Prince Sharma, Nancy George, Prakram Singh Chauhan, Neena Puri, Naveen Gupta

**Affiliations:** 1 Department of Microbiology, BMS Block, Panjab University, Chandigarh, India; 2 Department of Industrial Microbiology, Guru Nanak Khalsa College, City Centre Road, Yamunanagar, 135001 Haryana India

**Keywords:** Extracellular laccase, *Bacillus tequilensis* SN4, Thermo-alkali-stable laccase, Response surface methodology, Pulp biobleaching

## Abstract

Degradation of residual lignin in kraft pulp by chemical bleaching is implicated in causing environmental pollution. The use of thermo- and alkali-tolerant bacterial laccases is considered to be important biological alternative to chemical processing. Laccases from *Bacillus* species have shown promise in this respect but their intracellular/spore bound presence make their industrial application economically unfeasible. We report here on a novel extracellular active thermo-alkali-stable laccase (SN4 laccase)  which is active at 90 °C and pH 8.0 using 2,6-dimethoxyphenol as substrate from *Bacillus tequilensis* SN4. SN4 laccase retained 27 % activity for 5 min at 100 °C and more than 80 % activity for 24 h at 70 °C. The enzyme is also stable at a higher pH (9.0–10.0). Enzyme production was optimized by submerged fermentation. Relatively high yields (18,356 nkats ml^−1^) of SN4 laccase was obtained in a medium containing 650 μM MnSO_4_, 350 μM FeSO_4_, and 3.5 % ethanol. A 764-fold increase in laccase activity was observed under optimal conditions. In addition, reduction in kappa number and increase in brightness of softwood pulp by 28 and 7.6 %, respectively, were observed after treatment with SN4 laccase without a mediator. When *N*-hydroxybenzotriazole was used as a mediator, the kappa number was decreased to 47 % and brightness was increased to 12 %.

## Introduction

Laccases (benzenediol:oxygen oxidoreductases; EC 1.10.3.2) are multicopper oxidases, which catalyze the oxidation of a wide variety of organic and inorganic compounds with concomitant four-electron reduction of molecular oxygen to water. They catalyze the oxidation of both phenolic and non-phenolic substrates (Imran et al. 2012). In general, laccases oxidize phenols and aromatic amines such as methoxyphenols, phenols, polyphenols, anilines, aryl diamines, hydroxyindole, benzenethiols, and some cyanide complexes of metals such as [Mo(CN)_8_]^4−^, [Fe(CN)_6_]^4−^, [Os(CN)_6_]^4−^, and [W(CN)_8_]^4−^ (Santhanam et al. 2011). Laccases are very useful enzymes with respect to their applications in industry. They have found uses in biotechnological applications such as biobleaching, xenobiotics bioremediation, decolorization of textile dyes, biosensors, food industry, and plastic degradation (Shraddha et al. 2011).

Laccases are widely distributed in nature. They were first observed in the exudates of Japanese lacquer tree *Rhus vernicifera* (Yoshida 1883) and have henceforth been found in almost all spheres of life but have been most extensively studied in fungi including *Ascomycetes, Basidiomycetes,* and *Deuteromycetes* (Brijwani et al. 2010).

Till date, laccases from only a few bacteria (*Azospirillum lipoferum*, *Marinomonas mediterranea*, *Bacillus subtilis,* etc.) have been characterized (Sharma et al. 2007). Bacterial laccases have several properties that are not characteristics of fungal laccases such as stability at high temperature (Martins et al. 2002; Miyazaki 2005) and high pH (Singh et al. 2007), and salt tolerance (Ruijssenaars and Hartmans 2004). Also, the production of fungal laccases in large quantity is problematic due to the accumulation of fungal biomass. Still, the use of bacterial laccases could not be exploited on an industrial scale as most of the reported laccases in bacteria are intracellular or spore bound, which makes their production and purification in large quantity extremely difficult. However, analysis of several bacterial genomes showed that there are 76 % of putative laccase genes that carry the signal sequences, which could enable them to be secreted outside the cytoplasm (Ausec et al. 2011). With this viewpoint in mind, we have designed the present study for the isolation of bacteria producing extracellular laccase stable at high temperature and high pH that is suitable for industrial applications.

Moreover, for hyperproduction of this enzyme to meet the industrial standards, an attempt was made to optimize the enzyme production. In addition to the conventional stepwise approaches, recent statistics-based experimental designs including Plackett–Burman (PB) and response surface methodology (RSM) have been used (Niladevi and Prema 2008). The ability of laccase to biobleach softwood pulp was also explored.

## Materials and methods

### Chemicals

Guaiacol, 2,2′-Azino bis(3-ethylbenzthiazoline-6-sulfonate) (ABTS), and syringaldazine (SGZ) were purchased from Sigma (USA). Other chemicals were obtained from Himedia (India) and were of an analytical grade. Unbleached softwood sulfite pulp (Kappa number 12.71 and brightness 25) used in this study was provided by Ballarpur Industries limited, Yamunanagar, Haryana, India.

### Laccase assay

The enzyme assay was performed at 90 °C for 5 min in 0.1 M phosphate buffer (pH 8.0) using 2,6-dimethoxyphenol (2 mM) (DMP) as substrate. The change in absorbance due to the oxidation of DMP was monitored at 470 nm (*ε* = 14,800 M^−1^ cm^−1^) in a UV–Visible spectrophotometer. The enzyme unit was expressed in nanokatals ml^−1^. One nkat of enzyme activity was defined as nanomoles of substrate converted into product per second by one ml of enzyme.

### Isolation of laccase-producing bacteria

Laccase-producing bacteria were isolated on M162 medium (Degryse et al. 1978) supplemented with 0.2 % yeast extract, 0.2 % tryptone, 100 μM CuSO_4_, and 2 mM guaiacol. The sludge samples from effluent treatment plant of paper and textile industry and soil samples from the areas where wood was decaying were enriched and appropriate dilutions were plated. The plates were incubated at 37 °C for 48 h. The colonies showing reddish brown color were selected.

### Selection of bacterial strains producing extracellular laccase

Selected bacterial cultures were screened for the presence of laccase enzyme. For this, 0.1 % of overnight-grown bacterial culture was inoculated in M162 broth containing 0.2 % yeast extract, 0.2 % tryptone, and 100 μM CuSO_4_. After 48 h, the culture supernatant was obtained by centrifugation at 7,826×*g* for 15 min and was used as extracellular enzyme. Cells obtained were washed twice, resuspended in 5 ml of 0.1 M Tris–HCl buffer (pH 8.0), disrupted by ultrasonication, and centrifuged at 7,826×*g* for 10 min at 4 °C. The supernatant obtained from the cell extract was used as intracellular enzyme. The laccase activity was examined in both intracellular as well as extracellular enzyme preparations using DMP as substrate. The strains exhibiting extracellular laccase activities were selected. Furthermore, the oxidation of SGZ, ABTS, and tyrosine was also assayed to confirm the presence of true laccase activity.

### Time course studies of isolate SN4 for growth and enzyme yield

For analyzing the growth properties as well as laccase production, M162 medium was inoculated with 0.1 % of 24-h-old culture of isolate SN4 and incubated at 37 °C, 150 rpm for 0–120 h. After specific time intervals, optical density was monitored at 600 nm and appropriate dilutions of the culture were plated on M162 agar plates for colony-forming unit (cfu) counts. The culture was centrifuged at 7,826×*g* for 10 min, and the extracellular as well as intracellular laccase activity was assayed according to the standard assay conditions.

### Analysis to show the absence of laccase activity in spores

Spores of the isolate SN4 were obtained according to the method given by Schaeffer et al. (1965). The spores were harvested and suspended in 50 mM Tris–HCl buffer (pH 8.0), and laccase activity was assayed in spore suspension.

### Identification of the isolate

The morphology of the isolate was studied by Gram staining. Physiological and biochemical characterization was done according to Bergey’s Manual of Determinative Bacteriology (Holt et al. 1994). Furthermore, the organism was identified based on 16S rDNA sequence, wherein the genomic DNA was isolated and 16S rDNA fragment was amplified. The product was sequenced using universal primers. Sequences homologous to isolate SN4 were obtained using EzTaxon. Sequences with high query coverage and homology were selected for phylogenetic analysis (Chun et al. 2007). Multiple sequence alignment was done using MultAlin (version 5) (Corpet 1988), and phylogenetic tree was constructed using MEGA version 4.0 (Tamura et al. 2007).

### Partial purification of SN4 laccase

The enzyme was partially purified by acetone precipitation. Chilled acetone (−20 °C) at a concentration of 60 % was added to crude enzyme preparation, kept for 2−3 h at −20 °C, and then centrifuged at 7,826×*g* for 15 min at 4 °C. The supernatant was discarded, and pellet was kept at room temperature till the residual acetone evaporated. It was then dissolved in 0.1 M Tris–HCl buffer (pH 8.0) (Adegoke et al. 2012).

### Effect of temperature and pH on laccase activity and stability

Effect of temperature on laccase activity was determined by performing enzyme assay at different temperatures (55–100 °C). Thermostability of enzyme was measured over the temperature range of 65–100 °C by incubating the enzyme in thin-wall test tubes for 0–4 h. At different time intervals, aliquots of enzyme were taken and chilled in an ice-water bath. The residual enzyme activity was determined as per standard assay procedure.

The optimum pH for laccase activity using DMP (2 mM) as substrate was determined by performing the enzyme assay at different pH values ranging from 6.0 to 7.5 (0.1 M phosphate buffer), 8.0 to 9.0 (0.1 M Tris–HCl buffer), and 9.5 to 10.0 (0.1 M carbonate–bicarbonate buffer). pH stability of SN4 laccase was measured over a pH range of 7.0–10.0 by incubating the enzyme in buffers of various pH for 0–3 h at room temperature. At different time intervals, aliquots of enzyme were taken and residual enzyme activity was determined at 90 °C as per protocol.

## Optimization of SN4 laccase production in submerged fermentation

### Optimization by one variable at a time method (OVAT)

For increasing the laccase production from *Bacillus tequilensis* SN4, optimization of various nutritional and environmental parameters including incubation time, temperature, pH, different concentrations of CuSO_4_ (0–1,000 μM), different nitrogen sources, inoculum size, agitation rates (0–250 rpm), and metal ions was carried out by classical OVAT method. All the experiments were carried out at 30 °C and 150 rpm for 96 h.

### Experimental design of Plackett–Burman

In order to screen the key factors having significant influence on laccase production, a Plackett–Burman experimental design was formulated with nineteen parameters, based on the literature search and OVAT results. The parameter evaluated was as follows: *A*: CuSO_4_, *B*: MnSO_4_, *C*: MgSO_4_, *D*: FeSO_4_, *E*: CoSO_4_, *F*: Al_2_(SO_4_)_3_, *G*: yeast extract, *H*: wheat bran, *J*: 2,6-Xylidine, *K*: pyrogallol, *L*: catechol, *M*: ferulic acid, *N*: tryptone, *O*: vanillin, *P*: vanillic acid, *Q*: methanol, *R*: ethanol, *S*: phenol, and *T*: (NH_4_)_2_SO_4_. Each factor was investigated at two levels, high (+) and low (−). A design of total 20 experiments was generated by using the software Design Expert 8.0.7.1. The average of enzyme activity obtained was taken as response. The effect of individual factors on enzyme activity was calculated according to the following equation:

1 $$E_{i}=\frac{\left(\Sigma P_{i+}-\Sigma P_{i-}\right)}{N}$$

where *E*_*i*_ is the effect of parameter *i* under study, *P*_*i+*_ and *P*_*i−*_ are responses (enzyme activity) of trials at which the parameter was at its high and low level, respectively, and *N* is the total number of trials. From the Pareto chart, the factors showing highest positive effects were selected for optimization using central composite design (CCD) of RSM.

### CCD of RSM

The three most significant variables chosen from the PB were optimized through CCD of RSM, while the other significant factors were fixed at a constant level (same as in the basal medium). For three variables, MnSO_4_, FeSO_4_, and ethanol, there were six central, eight factorial, and six axial points in CCD. The response was fitted by a second-order model to be correlated with the independent parameters. The correlation between the three parameters and the response (laccase activity) was described by the following predictive quadratic polynomial equation:

2 $$Y\;=\;\beta_{0}+\;\Sigma \;\beta_{i}X_{i}+\;\Sigma \;\beta_{ii}X_{i2}+\;\Sigma \;\beta_{ij}X_{i}X_{j}$$

where *Y* is the predicted response [laccase activity (nkats ml^−1^)], *β*_0_ is the constant term, *β*_*i*_ the linear coefficients, *β*_*ii*_ the squared coefficients, and *β*_*ij*_ the interaction coefficients. The quality of fitting by the polynomial model equation was expressed using coefficient of determination *R*^2^. Equation 2 was used to construct 3D plots.

The experimental plan consisted of 20 trials, and the three variables viz. MnSO_4_, FeSO_4_, and ethanol were studied at three different levels, low (−1), medium (0), and high (+1). The optimal values of the three parameters were achieved by solving the obtained polynomial equation. In addition to it, three-dimensional plots were constructed for visual observation of the trend of maximum response and the interactive effects of the significant variables on the response.

### Validation of the model

The RSM model was validated further for predicted versus actual responses. Each experiment was carried out in triplicate, and the results were compared with the predicted responses.

### Biobleaching of softwood pulp

Softwood pulp procured from Ballarpur Industries Limited (BILT), Yamunanagar, Haryana, India, was washed with distilled water and dried at 55 °C in an oven overnight. Pulp with 5 % consistency was treated with 40 U g^−1^ oven-dried pulp of laccase enzyme at a temperature of 65 °C and pH 9.0 for 4 h (conditions standardized in our laboratory). Pulp treated under the same conditions but without enzyme was taken as control. One set of experiment was done without any mediator, and the other set was done using 2 mM *N*-hydroxybenzotriazole (HOBT) as mediator. Both the control and enzyme-treated pulp samples were filtered through muslin cloth, washed with distilled water, and then dried in oven at 55–60 °C overnight. Kappa number and brightness of pulp were determined according to the TAPPI method (Technical Association of pulp and paper industry, Atlanta, USA) T 236 and T 452, respectively.

### Statistical analysis

All the experiments were carried out in triplicate, and the mean ± SD has been reported. Data were analyzed using analysis of variance (ANOVA) by Sigma Stat version 2.03 and values that were statistically significant (*p* < 0.05) were taken.

## Results

### Isolation and selection of extracellular laccase-producing bacteria

Ten bacterial isolates producing laccase were selected from the sludge samples of paper mill. These isolates were examined for extracellular and intracellular laccase activity. Isolate number 4 was found to produce an extracellular laccase, which was able to oxidize laccase-specific substrates, SGZ and ABTS, but not tyrosine. This isolate was selected and designated as isolate SN4.

**Fig. 1:**
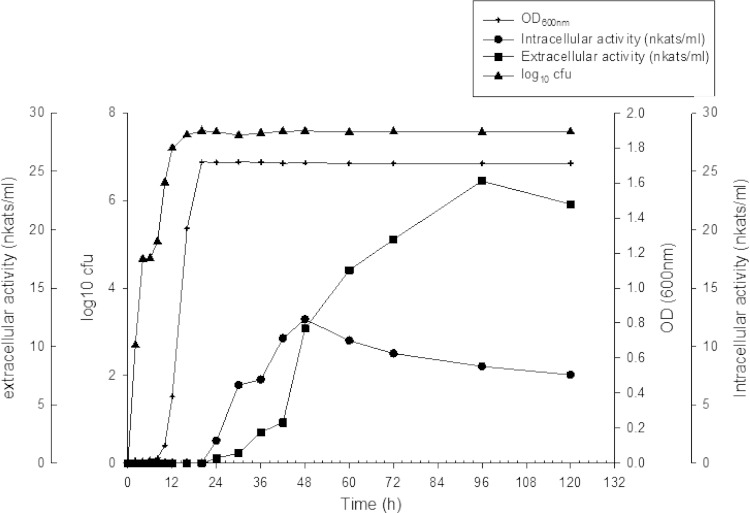
Growth profile of *B. tequilensis* SN4 showing colony-forming unit (cfu), optical density (OD) at 600 nm, extracellular and intracellular enzyme production

The growth and enzyme production profile of SN4 was studied over a period of 120 h. The expression of laccase inside the cells started at the onset of stationary phase. Extracellular laccase activity was detected in the culture supernatant after 24 h, while no lysis of bacterial cells was observed up to 96 h (Fig. 1). No laccase activity was detected in isolate SN4 spore suspension.

### Identification of the isolate

On morphological and biochemical analysis, isolate SN4 was found to be similar to the organism of Genus *Bacillus*. On 16S rDNA analysis, maximum similarity (99.93 %) (GenBank accession number KF150708) was obtained with *B. tequilensis* 10b^T^ (Gatson et al. 2006). However, SN4 was different from *B. tequilensis* 10b^T^ in some of the biochemical characteristics (oxidase, citrate, xylose, arginine dihydrolase, and Voges–Proskauer test). Therefore, it was concluded that the isolate SN4 could be a new strain of *B. tequilensis.* In this study, the isolate was designated as *B. tequilensis* strain SN4 (MTCC No. 11828).

### Temperature and pH optima and stability

**Fig. 2:**
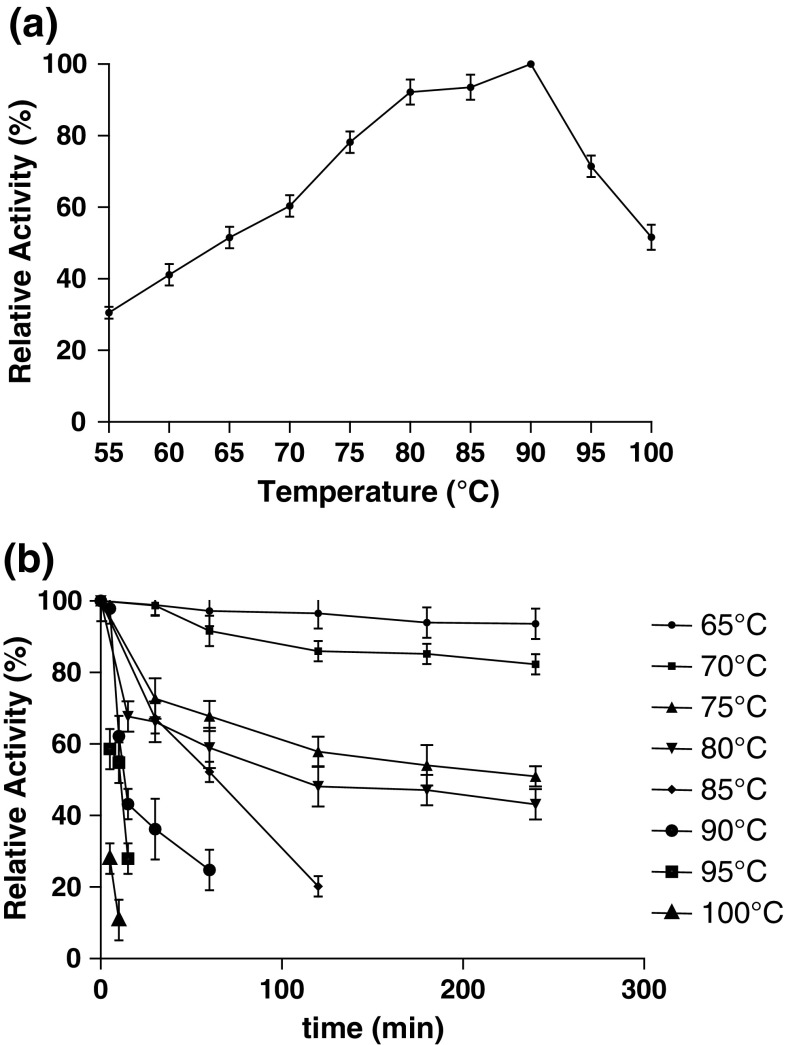
Effect of temperature on laccase enzyme activity **a** optimum temperature of laccase activity **b** stability of enzyme at various temperatures

The optimum temperature at which SN4 laccase was found to be active was in the range of 80–90 °C, maximum being at 90 °C (Fig. 2a). SN4 laccase retained 59 and 27 % activity even at 95 and 100 °C, respectively, after 5 min of the incubation at these temperatures (Fig. 2b). It was found to be more than 80 % stable at 70 °C for 24 h (data not shown).

**Fig. 3:**
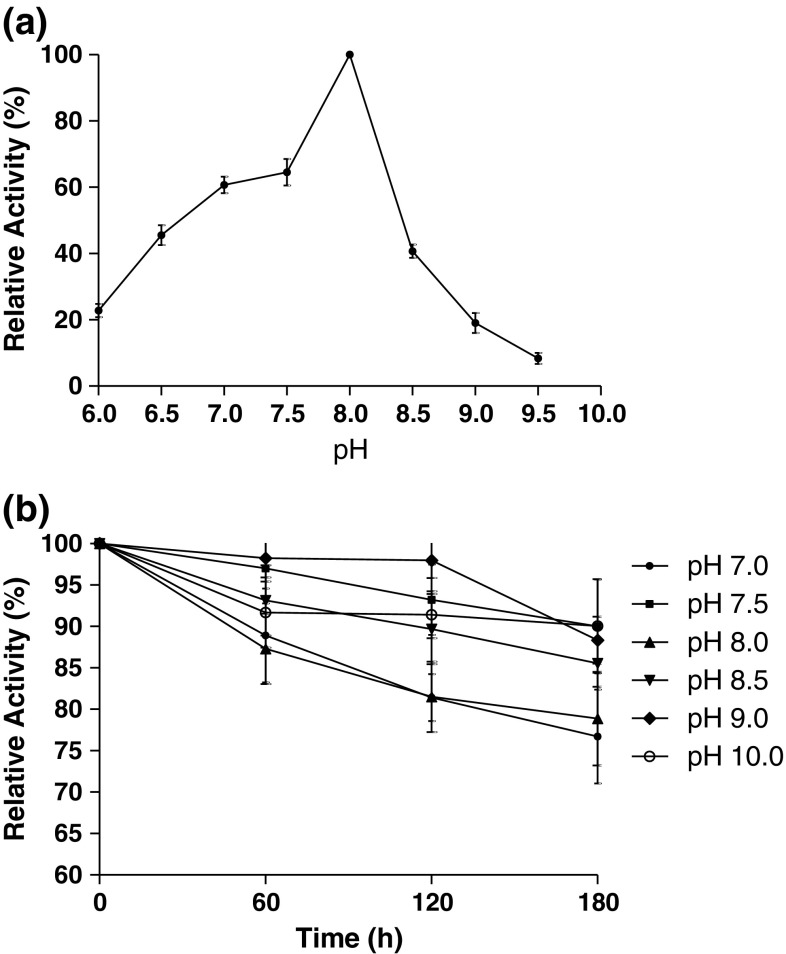
Effect of pH on laccase enzyme activity **a** optimum pH **b** stability of laccase at different pH values

The optimum pH of SN4 laccase was found to be 8.0 (Fig. 3a). Moreover, this enzyme was highly alkali-stable as it retained 80–90 % of initial activity after 3-h incubation at pH 7.0–10.0 (Fig. 3b).

## Optimization of enzyme production

### OVAT method

In order to increase the yield of laccase, its production was optimized by classical one variable at a time method (Table 1). A remarkable increase in the enzyme activity, i.e., from 24 to 1,514 nkats ml^−1^, was achieved under following optimized conditions viz. 96-h incubation time, 30 °C temperature, pH 8.0, 100 μM CuSO_4_, 0.6 % yeast extract, 0.2 % tryptone, 150 rpm agitation rate, 0.3 % inoculum, and 300 μM MnSO_4_.

**Table 1:** Optimization of laccase from *B. tequilensis* SN4 by OVAT method

Parameter	Range	Optimum range	Optimum activity (nkats ml^−1^)
Time	0–120 h	96 h	24 ± 2.07
pH	6.0–10.0	8.0	106 ± 1.38
Temperature	25–42 °C	30 °C	124 ± 1.84
CuSO_4_	0–1,000 μM	100 μM	130 ± 2.58
Yeast extract	0.2–1.2 %	0.6 %	229 ± 0.89
Inoculum	0.05–1.0 %	0.3 %	240 ± 3.75
Agitation	0–250 rpm	150 rpm	245 ± 2.94
MnSO_4_	0–1,000 μmol	300 μM	1,514 ± 1.25

Values represent mean ± SD (*n* = 3)

### Screening the significant parameters for laccase production by PB analysis

**Fig. 4:**
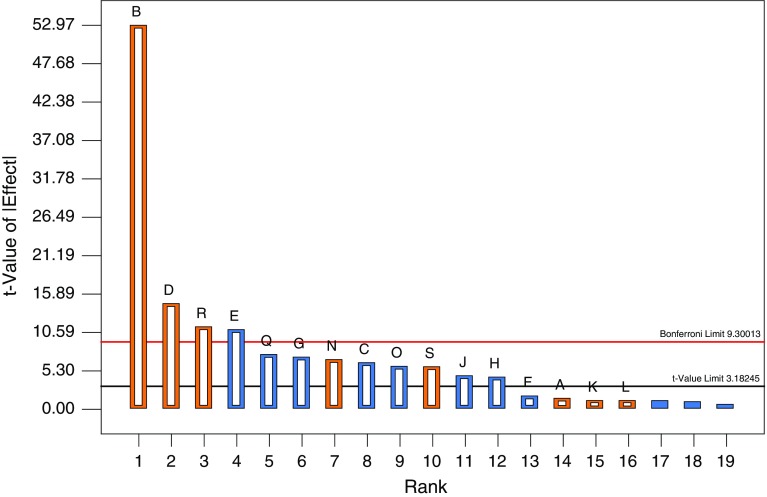
Pareto chart showing significant factors (*yellow color*) and nonsignificant factors (*blue color*)

The influence of various parameters on enzyme activity was estimated and graphically represented in the form of Pareto chart (Fig. 4). Length of column represented the significance of the influence of studied parameters on enzyme activity. Out of 19 different factors, 8 factors viz. MnSO_4_, FeSO_4_, ethanol, tryptone, phenol, CuSO_4_, pyrogallol, and catechol showed positive effects. Out of these, MnSO_4_ had highest influence on enzyme activity. When the value of the effect *E*_*i*_ of the parameter in question was positive, the enzyme activity was more at the higher level of the parameter. However, if the value was negative, the enzyme activity was greater at the lower level of the parameter. The above stated eight factors had positive influence on enzyme activity, which indicated that the laccase production could be enhanced by these factors. Therefore, in order to obtain the maximum enzyme production, optimal concentration of MnSO_4_, FeSO_4_, and ethanol would be investigated with RSM.

### Optimization of enzyme production through RSM

**Fig. 5:**
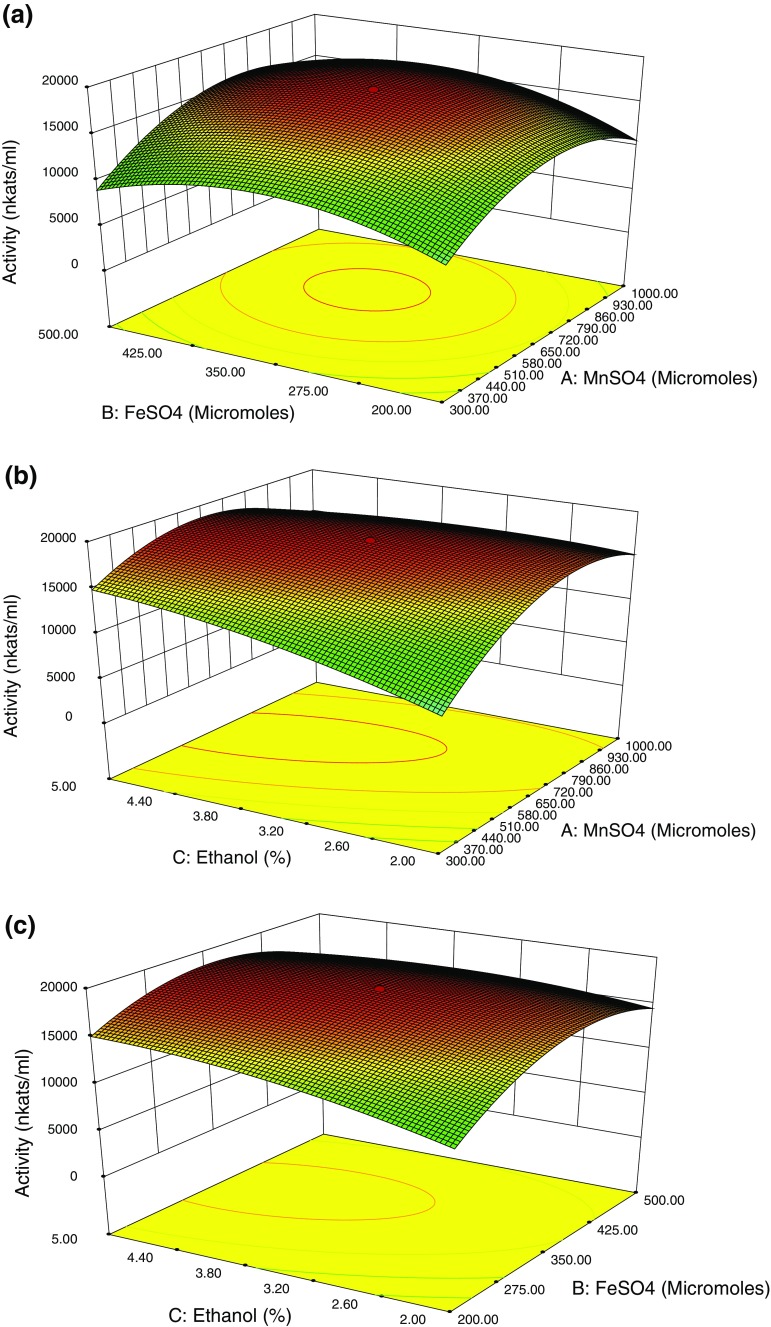
Three-dimensional response surface plots showing the effect of interaction of **a** MnSO_4_ and FeSO_4_**b** FeSO_4_ and ethanol **c** Ethanol and MnSO_4_ on laccase production from *B. tequilensis* SN4

Based on the PB test, MnSO_4_, FeSO_4_, and ethanol were identified as significant parameters, which enhanced the enzyme yield. Therefore, a CCD was formulated to investigate the optimum levels of these parameters. The experimental design and the response (enzyme activity) are presented in Table 2. By applying multiple regression analysis on the experimental data, a predictive quadratic polynomial equation was constructed to describe the correlation between enzyme activity and the three significant parameters as follows:

$$R\;=\;+16,696.86\;+\;1,819.23\;\times \;A\;+\;982.91\;\times \;B\;+\;1,546.09\;\times \;C\;+\;466.63\;\times \;A\;\times \;B\;-\;1,829.12\;\times \;A\;\times \;C\;-\;1,175.37\;\times \;B\;\times \;C\;-\;3,702.95\;\times \;A^{2}\;-\;2,829.49\;\times \;B^{2}\;-\;371.94\;\times \;C^{2}$$

where *R* is the response (laccase activity); *A*, *B*, and *C* were the coded values of MnSO_4_, FeSO_4_, and ethanol concentration, respectively. The analysis of variance for the response surface quadratic model is summarized in Table 3. The *p* < 0.0001 indicated that the linear, interactive, and squared terms all had quite significant influence on enzyme activity. The *p* value for the lack of fit was 0.6148, indicating that this quadratic model adequately fit into the data. The determination coefficient *R*^2^ (0.9958) indicated that the predicted and experimental values had perfect coherence with each other. The value of adjusted *R*^2^ (0.9921) suggested that the variation of 99.21 % in the enzyme activity was attributed to the independent variables and only 0.79 % of the total variation could not be explained by the model. The maximum enzyme activity of 18,245 nkats ml^−1^ was obtained when 650 μM MnSO_4_, 350 μM FeSO_4_, and 3.5 % ethanol were added to the medium. It was observed from 3D curves (Fig. 5) that the maximum response was located inside the design boundary, which validated tested ranges of the parameters. The shape of the curve indicated whether the interactions between the factors were significant or not. The spherical curves in Fig. 5a–c indicated that the interactions between MnSO_4_, FeSO_4_, and ethanol were significant. According to Fig. 5, the relative significance of the impact of the parameters on enzyme activity was in the following order: MnSO_4_ > FeSO_4_ > ethanol, which was in accordance with the results obtained from Fig. 4. Therefore, this model could be used for the prediction of laccase activity from *B. tequilensis* SN4.

**Table 2:** Central composite design with predicted and actual responses

Run order	MnSO_4_ (μM)	FeSO_4_ (μM)	Ethanol (%)	Predicted value (nkats ml^−1^)	Actual value (nkats ml^−1^)	Residual activity
1	1,000	500	2.0	14,643.72	14,935 ± 50.97	291.28
2	300	500	5.0	10,641.03	10,845 ± 35.47	203.97
3	650	350	3.5	18,174.66	18,222 ± 48.13	47.34
4	300	200	5.0	11,812.77	11,843 ± 37.63	30.23
5	1,000	200	5.0	10,139.39	11,174 ± 30.17	34.61
6	1,000	200	2.0	9,247.46	9,365 ± 39.58	117.54
7	650	350	3.5	18,174.66	18,245 ± 27.54	70.34
8	1,000	500	5.0	11,334.16	11,509 ± 31.78	174.84
9	650	350	3.5	18,174.66	18,152 ± 25.06	−22.66
10	650	350	3.5	18,174.66	18,156 ± 37.31	−18.66
11	300	500	2.0	6,134.09	6,421 ± 38.69	286.91
12	300	200	2.0	3,104.33	3,251 ± 39.61	146.67
13	650	97.73	3.5	6,967.65	6,927 ± 25.02	−40.65
14	650	602.27	3.5	10,520.04	10,106 ± 22.47	−414.04
15	650	350	3.5	18,174.66	18,239 ± 30.92	64.34
16	1,238.63	350	3.5	9,297.63	9,085 ± 25.65	−212.63
17	650	350	0.98	12,924.89	12,579 ± 25.10	−345.89
18	61.37	350	3.5	3,549.06	3,007 ± 42.50	−242.06
19	650	350	3.5	18,174.66	18,112 ± 35.79	−62.66
20	650	350	6.02	17,464.79	18,356 ± 27.30	−108.79

Values represent mean ± SD (*n* = 3)

**Table 3:** Analysis of variance of response surface methodology

Source	Sum of squares	*df*	Mean square	*F* value	*p* value prob > *F*	
Model	4.171E+	9	4.635E+7	264.53	<0.0001	Significant
*A*	4.520E+7	1	4.520E+7	257.98	<0.0001	
*B*	1.319E+7	1	1.319E+7	75.31	<0.0001	
*C*	3.265E+7	1	3.265E+7	186.33	<0.0001	
*AB*	1.742E+6	1	1.742E+6	9.94	0.0103	
*AC*	2.677E+7	1	2.677E+7	152.77	<0.0001	
*BC*	1.105E+7	1	1.105E+7	63.08	<0.0001	
*A* ^2^	1.976E+8	1	1.976E+8	1,127.88	<0.0001	
*B* ^2^	1.154E+8	1	1.154E+8	658.54	<0.0001	
*C* ^2^	1.994E+6	1	1.994E+6	11.38	0.0071	
Residual	1.752E+6	10	1.752E+5			
Lack of fit	7.564E+5	5	1.513E+5	0.76	0.6148	Not significant
Pure error	9.956E+5	5	1.991E+5			
Cor total	4.189E+8	19				
Model fitting	C.V = 3.49 %	*R*^2^ = 0.9958		*R*^2^ (pred) = 0.9921	*R*^2^ (adj) = 0.9824	

*A* MnSO_4_, *B* FeSO_4_, *C* ethanol

### Validation of the mathematic model

In order to validate the predicted results of the quadratic model, the cultivation of *B. tequilensis* SN4 was carried out under predicted optimal conditions in triplicates. The average enzyme activity of 18,356 nkats ml^−1^ (~1,101 U ml^−1^) was obtained under optimized conditions, which was very close to the predicted value (18,174). The coherence between the experimental and estimated responses verified the validity and accuracy of the model in terms of predicting the enzyme production by *B. tequilensis* SN4. The enzyme activity obtained was 764-folds higher than the one obtained under unoptimized conditions.

### Reduction in kappa number of softwood pulp with SN4 laccase

Laccase from *B. tequilensis* SN4 was applied for the biobleaching of softwood pulp. The enzyme showed 28 % reduction in kappa number and 7.6 % increase in brightness without any mediator. The use of 2 mM HOBT as mediator further improved the biobleaching by reducing the kappa number to 47 % and increasing the brightness to 12 % (Table 4).

**Table 4:** Physicochemical properties of softwood pulp treated with laccase from *B. tequilensis* SN4

	Kappa number		Brightness	
	Kappa number	% Reduction	Brightness (% ISO)	% Increase
Control^a^	12.71 ± 0.29	–	25.00 ± 0.056	0
Laccase treated	9.147 ± 0.21	28.05	26.00 ± 0.028	7.69
LMS^#^	6.701 ± 0.28	47.29	28.00 ± 0.016	12.00

Values represent mean ± SD (*n* = 3)

^*#*^Laccase mediator system

^a^The control pulp was treated under same conditions with buffer but without enzyme

## Discussion

Most of the bacterial laccases reported so far are either intracellular or a component of bacterial spore coat protein Cot A (Sharma et al. 2007), which makes the application of bacterial laccases problematic at industrial scale. However, in a recent study on the diversity of laccase in bacterial genome, most of the potent laccase genes have been shown to carry signal sequences required for the secretion of enzyme into extracellular milieu (Ausec et al. 2011). In the present study, *B. tequilensis* SN4 was isolated from the activated sludge of paper mill effluent treatment plant, which was found to produce an extracellular laccase. In order to prove that SN4 laccase is a true extracellular enzyme and does not appear in the medium due to cell lysis, as reported in the case of γ-proteobacterium JB (Singh et al. 2007), the growth and enzyme production profile of *B. tequilensis* SN4 was studied with respect to time. It was observed that extracellular laccase production started after 24 h while no lysis of bacterial cells was observed up to 120 h. Moreover, no laccase like activity was observed in the spores as reported in other *Bacillus* spp. (Martins et al. 2002; Ruijssenaars and Hartmans 2004).

Some enzymes like tyrosinase have an overlapping substrate range with laccases. SGZ and ABTS are laccase-specific substrates. The ability of an enzyme to oxidize SGZ and ABTS, with an inability to oxidize tyrosine, is an indicator of true laccase activity (Williamson 1994). SN4 laccase was found to oxidize SGZ and ABTS but not tyrosine. This confirms that the laccase from SN4 is a true laccase. In contrast, the only other extracellular laccase from *Bacillus* sp. ADR is unable to oxidize SGZ and ABTS indicating that it is not a true laccase (Telke et al. 2011).

Besides being extracellular, some other properties of enzymes like stability at high temperature and pH values are required for it to be exploited on industrial scale. SN4 laccase was found to have optimum activity at 90 °C and was also found to be stable at high temperature (>80 % at 70 °C for 24 h). Moreover, the laccase from *B. tequilensis* SN4 is also stable at high pH. To the best of our knowledge, no other extracellular bacterial laccase has been reported with such high thermo-alkali stability. These properties also make SN4 laccase an important biomolecule for structural and functional studies.

For industrial applications, laccase production was optimized by classical OVAT and by statistical designs including PB and RSM. A 63.08-fold increase in laccase activity was observed with OVAT method. In PB, out of 19 different parameters tested, eight were found to have positive effect on laccase production. Out of these, MnSO_4_ followed by FeSO_4_ and ethanol were having maximum effect on enzyme production. Gene expression of laccase has been reported to be regulated mainly by Cu^2+^ but other metal ions such as Mn^2+^, Fe^2+^, and ethanol have also been recognized as potent inducers of laccase (Gonzalez et al. 2013). Mn^2+^ has been reported to be an effective inducer of laccase in case of *Pluerotus sajor*-*caju* (Soden and Dobson 2001) and *Coprinus comatus* (Lu and Ding 2010). Laccase production from *C. comatus* has been increased from 50 to 225 U (4.5-fold increase) by supplementation of 0.8 mM Mn^2+^ in the medium (Lu and Ding 2010). Different studies have shown that laccase production is regulated by metal ions such as Cu^2+^, Fe^2+^, and Mn^2+^ by gene expression induction or through translational or posttranslational regulation (Soden and Dobson 2001; Fonseca et al. 2010). Increase in laccase production from *B. tequilensis* SN4 by metal ions in this study can be because of similar reasons. The positive influence of organic solvents on laccase production has also been reported (Lee et al. 1999; Dhakar and Pandey 2013). Addition of ethanol to the production media increased the laccase production by 65-folds in *Pycnoporus cinnabarinus* (Lomascolo et al. 2003). These three variables were chosen to further optimize laccase production through RSM. A 764-fold increase in laccase production was observed in comparison with the unoptimized value. This enzyme yield is higher than the other reported laccases not only from bacteria but also from fungi. The use of MnSO_4_, FeSO_4_, and ethanol in place of known laccase inducers like veratryl alcohol and ferulic acid makes the laccase production from *B. tequilensis* SN4 both economically as well as ecologically significant.

Laccases have natural tendency to degrade lignin (Virk et al. 2012). As SN4 laccase is extracellular, thermostable, alkali-stable, and can be produced economically in large quantities, it can be a useful candidate for application in pulp biobleaching. The use of partially purified SN4 laccase was explored for biobleaching of softwood pulp. The enzyme showed significant reduction in kappa number and increase in brightness with as well as without mediator. Reduction in kappa number without using mediator further makes SN4 laccase an attractive candidate for application in pulp biobleaching.

## Conclusion

It can be concluded from the present study that *B. tequilensis* SN4 produces a novel, highly thermostable and alkali-stable extracellular laccase. The production of SN4 laccase has been optimized to give higher yield at significantly lower cost. Moreover, the enzyme is capable of making significant reduction in kappa number and increase in the brightness of pulp even without the use of mediator, thus making the process industrially viable. 
